# Blood Pressure Signal Entropy as a Novel Marker of Physical Frailty: Results from the FRAILMatics Clinical Cohort

**DOI:** 10.3390/jcm12010053

**Published:** 2022-12-21

**Authors:** Silvin P. Knight, Eoin Duggan, Roman Romero-Ortuno

**Affiliations:** 1 Discipline of Medical Gerontology, School of Medicine, Trinity College Dublin, D02 R590 Dublin, Ireland; 2 Falls and Syncope Unit (FASU), Mercer’s Institute for Successful Ageing (MISA), St. James’s Hospital, D08 E191 Dublin, Ireland

**Keywords:** sample entropy, physical frailty, cardiovascular, blood pressure, clinical validation

## Abstract

In this study we investigated the association between information entropy in short length blood pressure signals and physical frailty status, in a group of patients aged 50+ recruited from the Falls and Syncope Unit at the Mercer’s Institute for Successful Ageing in St James’s Hospital, Dublin, Ireland. This work is an external clinical validation of findings previously derived in a population-based cohort from The Irish Longitudinal Study on Ageing (TILDA). The hypothesis under investigation was that dysregulation (as quantified by entropy) in continuous non-invasive blood pressure signals could provide a clinically useful marker of physical frailty status. We found that in the 100 patients investigated, higher entropy in continuously measured resting state diastolic blood pressure was associated with worse physical frailty score, as measured by the Frailty Instrument for primary care of the Survey of Health, Ageing and Retirement in Europe (SHARE-FI). Since physical frailty is defined as a pre-disability state and hence it can be difficult for clinicians to identify at an early stage, the quantification of entropy in short length cardiovascular signals could provide a clinically useful marker of the physiological dysregulations that underlie physical frailty, potentially aiding in identifying individuals at higher risk of adverse health outcomes.

## 1. Introduction

Frailty is a biologically driven decrease in reserve and resistance to stressors, resulting from collective declines across multiple physiological systems [1]. Frailty increases an individual’s vulnerability to adverse outcomes such as falls, hospitalization, institutionalization, and mortality [2,3,4,5]. For many older people, frailty can lead to loss of independence and impair quality of life and psychological wellbeing. Since many frailty operationalization schemes exist, the prevalence of frailty in community-dwelling European older adults varies between less than 5% and over 40% [6,7]. However, the physical frailty phenotype originally proposed by Fried et al. [2] is more specific than other classifications and its prevalence in Ireland in community-dwelling adults aged 50 years or over has been estimated at 3.8% [8].

The main advantage of Fried’s method is that it only requires the measurement of five variables, namely *unintentional weight loss, exhaustion* symptoms, *grip strength, walking speed* and self-reported *physical activity*. In this method, frailty is conceptualized as a pre-disability state [9] and quantified in terms of three categories (*non-frail*, *pre-frail*, or *frail*), each of which is simply defined by the sum of the number of dichotomized individual criteria present (0: non-frail, 1 or 2: pre-frail, and 3 or more: frail). One limitation with this approach is that it retrospectively dichotomizes criteria that are measured on a continuous scale (i.e., grip strength, walking speed and physical activity). In a 2010 study by Romero-Ortuno et al., this limitation was addressed using data from the Survey of Health, Ageing and Retirement in Europe (SHARE), a large (*N* > 31,000) population-based survey conducted in 2004–2005 in twelve European countries [10]. In that study, a data-driven approach was taken to construct the *SHARE Frailty Instrument for primary care* (SHARE-FI), which uses the 5 adapted physical frailty phenotype criteria, but by using a data weighing approach, also allows for the extraction of a continuous frailty score. Others have shown that the SHARE-FI continuous score provides stronger agreement, compared with the categorical scores, with a variety of validated physical performance measures in older adults [11,12]. In this study, the continuous SHARE-FI score was used to quantify physical frailty.

With the rising costs of health and social care due to population ageing worldwide, many countries are focusing health policies on disability prevention and health promotion among older individuals. Because frailty by phenotype is a pre-disability state, its early stages (e.g., pre-frailty) can be clinically difficult to recognize, especially as regards the presence of early underlying physiological dysregulations that may already be present in older individuals. Additionally, previous studies have shown that interventions can delay and even reverse frailty, especially when it presents in the early stages [13,14]. With this in mind, understanding the physiology underpinning the physical frailty syndrome, and identifying at-risk groups earlier, is of primary interest. Dysregulation of the cardiovascular system is thought to be associated with risk of frailty [15,16,17,18], and frailty may be both a cause and a consequence of cardiovascular disease [19]. One method to quantify disorder in continuous physiological signals is by means of information entropy [20,21]. Signal entropy quantifies the amount of irregularity or unpredictability in a particular dataset, assigning lower entropy values to periodic, predictable data, and higher entropy values to more irregular, unpredictable data.

Many different implementations of signal entropy have been proposed for the analysis of time-varying physiological signals, such as approximate entropy (ApEn) and sample entropy (SampEn) [20,21]. In the present work, we used SampEn to quantify disorder in continuously measured resting blood pressure time series data, since it overcomes some of the limitations of ApEn, namely the fact that self-matches are not counted and that resulting measures are largely independent of sample length [21]. Our team have previously demonstrated that higher signal entropy in blood pressure signals was associated with worse physical frailty status in a large population-based cohort of older individuals (*N* = 2645) from the Irish Longitudinal Study on Ageing (TILDA) [15], as well as worse cognitive performance, increased mortality risk, and increased risk of future falls and syncope [22,23,24]. However, results derived in this population-based cohort had not yet been validated in an external clinical sample. In TILDA, we found that a simple automated measure of cardiovascular signal complexity (entropy) could provide a clinically useful marker of the physiological dysregulations that underlie physical frailty; hence, the aim of the present study was to investigate whether this measure retained its utility in a much smaller (*N* = 100) pilot clinical sample of older patients aged 50 years and over attending an outpatient falls and syncope clinic.

## 2. Materials and Methods

### 2.1. Setting and Patients

We included a pilot sample of patients aged 50 years or older who attended the Falls and Syncope Unit (FASU) in St. James’s Hospital, Dublin, Ireland, between November 2021 and August 2022. FASU is a specialist outpatient clinic that uses non-invasive neuro-cardiovascular technologies to provide expert assessment of adult patients referred with dizziness, pre-syncope, syncope or falls [25]. Recruitment took place by one physician (E.D.) in FASU on average 2 mornings per week. To be included in the study, patients had to be age 50 years or over, and able to: give written informed consent, mobilize independently (with or without aid), and transfer (independently or with minimal assistance of one person) from lying to standing. Ethics approval for the study was granted from the Tallaght University Hospital/St. James’s Hospital Joint Research Ethics Committee (Project ID: 0221; approval date: 4 May 2021). Approval was also granted by St James’s Hospital Research & Innovation Office (Reference: 6567, approval date: 26 July 2021). All patients provided written informed consent. The study adhered to the World Medical Association Declaration of Helsinki on ethical principles for medical research involving human subjects.

### 2.2. Cardiovascular Measures

Participants began the assessment with the affixing of a digital photoplethysmograph to the index or middle finger of the left hand (Finapres^®^ NOVA, Finapres Medical Systems BV, Amsterdam, The Netherlands). Beat-to-beat blood pressure was measured at 200 Hz using the Finapres device. All measurements were carried out in a comfortably lit room, at an ambient temperature between 21 and 23 °C. Participants laid supine for 5–10 min and the last minute of resting state data was used for the entropy calculation. Signals for beat-to-beat systolic blood pressure (sBP) and diastolic blood pressure (dBP) data were extracted and linearly interpolated at 5 Hz using MATLAB (R2021b, The MathWorks, Inc., Natick, MA, USA).

### 2.3. Entropy Analysis

Entropy analysis was performed on the sBP and dBP data in MATLAB, using freely available code [26]. A detailed description of the algorithms used to compute SampEn has been previously reported in detail [21]; however, below we provide a brief overview.

For a timeseries of length *N*, $B_{i}^{m}\left(r\right)$ is defined as the number of template vectors of length *m*, $x_{m}\left(j\right)$, similar to $x_{m}\left(i\right)$ (within *r*) divided by *N* − *m* − 1, where *j* = 1 ... *N* − *m*, with *j* ≠ *i* (to avoid self-matches). The average $B_{i}^{m}\left(r\right)$ for all *i* is given as

(1) $$B^{m}\left(r\right)=\frac{1}{N-m}\overset{N-m}{\underset{i=1}{\sum }}B_{i}^{m}\left(r\right).$$

Similarly, we define $A_{i}^{m}\left(r\right)$ as the number of template vectors of length *m* + 1, $x_{m+1}\left(j\right)$, similar to $x_{m+1}\left(i\right)$ (within *r*) divided by *N* − *m* − 1, where *j* = 1 ... *N* − *m*, with *j* ≠ *i*. The average $A_{i}^{m}\left(r\right)$ for all *i* is given as

(2) $$A^{m}\left(r\right)=\frac{1}{N-m}\overset{N-m}{\underset{i=1}{\sum }}A_{i}^{m}\left(r\right).$$

SampEn was then calculated as

(3) $$\mathrm{SampEn}\left(m,r,N\right)=-\ln\left(\frac{A^{m}\left(r\right)}{B^{m}\left(r\right)}\right).$$

In this study, a range of *m* (embedding dimension; the length of the data segment being compared) values were investigated *(m* = [1, 2, 3, 4, 5]). Similarly, a range of *r* (similarity criterion) values were also investigated (*r* = [0.1, 0.15, 0.2, 0.25, 0.3, 0.35, 0.4, 0.45, 0.5]). SampEn was calculated for all potential combinations of *m* and *r*, for both sBP and dBP.

### 2.4. Physical Frailty Operationalization 

SHARE-FI [10] is an adapted physical frailty tool based on the following five measures:

- *Exhaustion* was identified as a positive response to the question: “In the last month, have you had too little energy to do the things you wanted to do?”. A positive answer (Yes) was coded as 1, and No was coded as 0.
- The *weight loss* criterion was fulfilled by reporting a “Diminution in desire for food” in response to the question: “What has your appetite been like?” or, in the case of a non-specific or uncodeable response to this question, by responding “Less” to the question: “So, have you been eating more or less than usual?”. The presence of the criterion was coded as 1 and its absence as 0.
- *Weakness* was assessed by handgrip strength, measured in kg using a Jamar Hydraulic Hand Dynamometer (Performance Health, Cedarburg, WI, USA). Two consecutive measurements were taken from the left and right hands, while seated. The maximum of the four attempts was used, providing a continuous variable.
- *Slowness* was defined as a positive answer to either of the following: “Because of a health problem, do you have difficulty [expected to last more than 3 months] walking 100 metres?” or “…do you have difficulty climbing one flight of stairs without resting?”. One or more positive responses was coded as 1, and two negative responses as 0.
- *Low activity* was assessed by the question: “How often do you engage in activities that require a low or moderate level of energy such as gardening, cleaning the car, or doing a walk?”. This resulted in an ordinal variable, where: 1 = “More than once a week”; 2 = “Once a week”; 3 = One to three times a month”; and 4 = “Hardly ever or never”.

The raw frailty score for an individual, *i*, was calculated as

SHARE-FI score(*i*) = *w*_fatigue_*z*_fatigue_(*i*) + *w*_weightloss_*z*_weightloss_(*i*) + *w*_weakness_*z*_weakness_ (*i*) + *w*_slowness_*z*_slowness_(*i*) + *w*_lowactivity_*z*_lowactivity_(*i*), (4)

where *z*_fatigue_(*i*) represents the standardized score on the fatigue measure for the individual *i* and *w*_fatigue_ is the sex-specific weighting associated with that measure (and similarly for the other four measures: weight loss, weakness, slowness, and low activity). Weighting values were taken from a previous study where these values were derived from a cohort of 31,115 individuals from 12 different European countries, using data from the Survey of Health, Ageing and Retirement in Europe (SHARE) [10]. Weighing values are provided in Appendix A, as well as sex specific cut-off for ‘non-frail’, ‘pre-frail’, and ‘frail’ categorization.

### 2.5. Other Measures

Variables that may have confounded the relationship between BP SampEn and SHARE-FI score were identified and included as covariates in some models. These included: age, sex, Cumulative Illness Rating Scale-Geriatric (CIRS-G) score, cardiovascular medication use, educational attainment, smoking status, alcohol consumption, and body mass index (BMI). Age and sex were recorded at the time of assessment. Disease status and medication use were obtained from participants’ self-report, medical chart review and/or correspondence from the participants’ General Practitioner if available. Educational attainment (primary vs. secondary/higher), smoking status (current vs. never/past), and alcohol consumption (units per week) were self-reported. The CIRS-G was used as a measure of extent and severity of morbidity in our sample [27]. The CIRS-G considers morbidities among 13 different organ systems and grades them from 0 (no problem) to 4 (severely incapacitating or life-threatening). Cardiovascular medication use was classified as ‘Yes’ if the patient was taking one or more of the following medications (coded using the Anatomical Therapeutic Chemical Classification (ATC)): anti-arrhythmics (ATC C01), anti-hypertensives (ATC C02), diuretics (ATC C03), vasodilators (ATC C04), beta-blocking agents (ATC C07), calcium channel blockers (ATC C08), or agents acting on renin-angiotensin system (ATC C09). Anthropometric measurements of height to the nearest 0.01 m (Seca 222 Stadiometer, Seca Ltd., Birmingham, UK) and weight to the nearest 0.1 kg (Seca 761 Mechnanical Scales, Seca Ltd., Birmingham, UK) were taken, and BMI was calculated from the formula weight [kg]/(height [m])^2^.

### 2.6. Statistical Analysis

Statistical analysis was performed using STATA 15.1 (StataCorp, College Station, TX, USA). Descriptive statistics were performed with calculation of the mean and standard deviation (SD) of continuous variables, and count and percentage of dichotomous variables. For sample description purpose, associations with the discrete ordinal SHARE-FI classification (i.e., non-frail, pre-frail and frail) were evaluated by means of the Spearman’s rank correlation coefficient (for continuous characterizing variables) and chi-square for trend (for categorical characterizing variables). Spearman’s rank correlation coefficient is a nonparametric measure of rank correlation which assesses how well the relationship between two variables can be described using a monotonic function; it is appropriate for both continuous and discrete ordinal variables and is not affected by the distribution of the data. Chi square test for trend is appropriate for testing the association between a nominal variable with two levels and an ordinal variable. Bivariate and multivariate robust linear regression models were computed to investigate the associations between BP SampEn (as independent variable) and the continuous SHARE-FI score (as dependent variable). Across the range of *r* and *m* values reported above, the bivariate relationships between BP SampEn and SHARE-FI score were calculated and explored graphically, and these results informed the choice of *r* and *m* used subsequently for the main models. Three sets of models were used: (1) unadjusted (bivariate), (2) adjusted for basic demographics and health measures (age, sex, CIRS-G score, and cardiovascular medication use), and (3) additionally adjusted for lifestyle and socioeconomic factors (age, sex, CIRS-G score, cardiovascular medication use, educational attainment, smoking status, alcohol consumption, and BMI). All entropy measures were standardized (z-scores). Normality of the residuals was assessed using normal quantile plots, standardized normal probability plots, and the skewness-kurtosis test for normality. Collinearity between model covariates was assessed via calculation of their variance inflation factors (VIF) in models (2) and (3) (VIF < 2: no collinearity). Results from absolute coefficients were reported as point estimates in appropriate units and presented with 95% confidence intervals (CI). Statistical significance was set at *p* < 0.05. To visualize the relationship between BP SampEn and SHARE-FI score, margin plots with 95% CI were also produced.

## 3. Results

### 3.1. Cohort Descriptives

Characteristics for the entire cohort, as well as those identified as non-frail, pre-frail, and frail by SHARE-FI, are given in Table 1. Increasing frailty category was associated with higher dBP SampEn and disease burden/severity, as well as higher proportions of women and current smokers.

### 3.2. Hyperparameter Tuning

**Figure A1:**
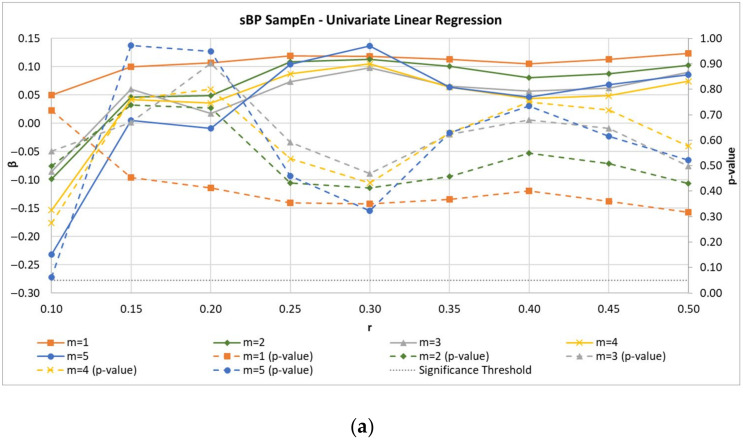

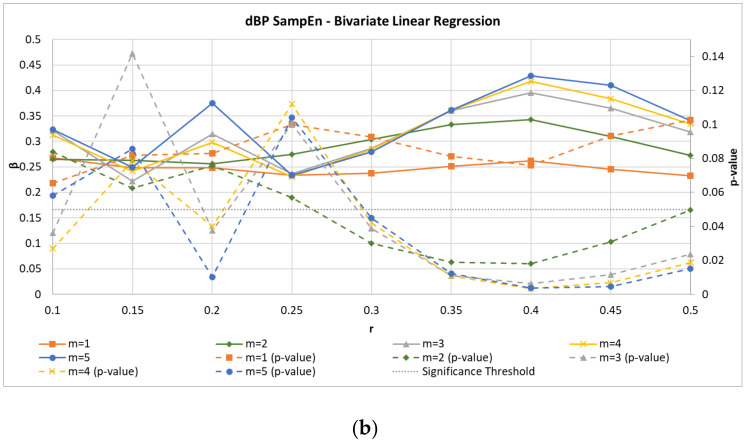
Results from the hyperparameter tuning using bivariate linear regression to investigate associations between the SHARE-FI continuous score and absolute SampEn values for (**a**) systolic blood pressure (sBP) and (**b**) diastolic blood pressure (dBP). The SampEn-associated values varied across *m* [1, 2, 3, 4, 5] and *r* [0.1, 0.15, 0.2, 0.25, 0.3, 0.35, 0.4, 0.45, 0.5] ranges. Solid lines show the resulting beta (*β*) coefficients and dashed lines show the associated *p*-values.

Results from the hyperparameter tuning are shown graphically as an Appendix to this work in Figure A1. Bivariate linear regression was used to investigate associations between the continuous SHARE-FI score and absolute SampEn values for sBP and dBP. The SampEn-associated values varied across *m* [1, 2, 3, 4, 5] and *r* [0.1, 0.15, 0.2, 0.25, 0.3, 0.35, 0.4, 0.45, 0.5] ranges. Solid lines show the resulting beta (*β*) coefficients and dashed lines show the associated *p*-values. Associations between the SHARE-FI score and sBP SampEn were not significant for any of the combinations of *m* and *r* investigated (see Figure A1a). Figure A1b revealed that for *r* < 0.3 association between the SHARE-FI score and dBP SampEn were mostly insignificant and did not seem to follow any trend. However, for *r* ≥ 0.3 all combinations of *r* and *m* (with the exception of *m* = 1) provided significant results. Since for dBP SampEn, *m* = 5 and *r* = 0.4 provided the largest *β* coefficient value and lowest *p*-value, these values were utilized for all subsequent models.

### 3.3. Regression Results

**Figure 1:**
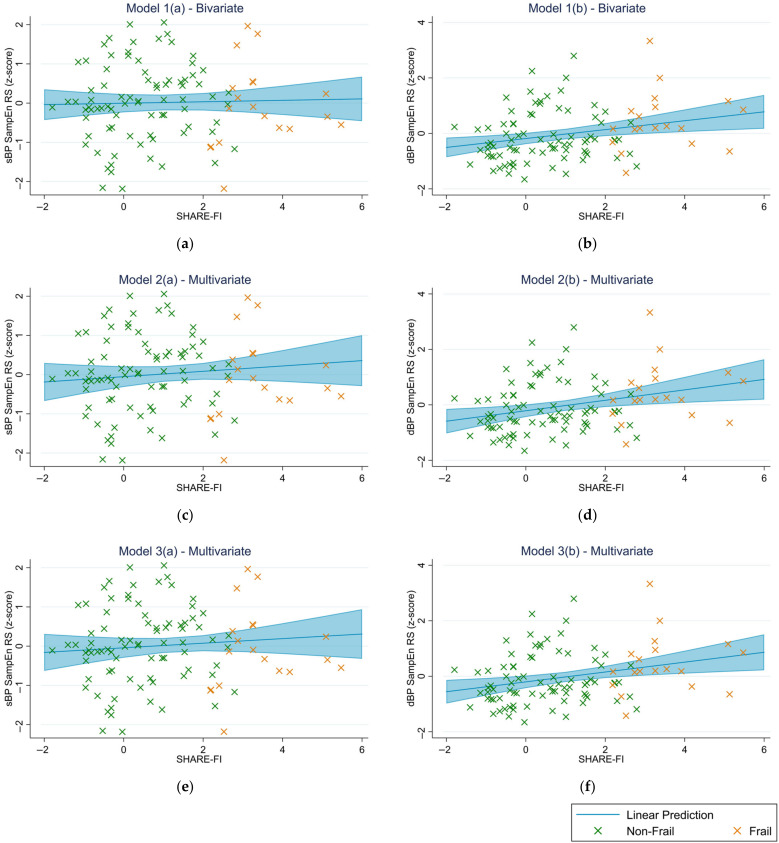
Margin plots illustrating the linear relationship between the continuous SHARE-FI score and BP SampEn (z-scored) from each of the models investigated. (**a**,**b**) show results from model 1, unadjusted (bivariate), for systolic blood pressure (sBP) and diastolic blood pressure (dBP) respectively. (**c**,**d**) show results from model 2, adjusted for age, sex, CIRS-G score, and cardiovascular medication use, for sBP and dBP respectively. (**e**,**f**) show results from model 3, adjusted for age, sex, CIRS-G score, cardiovascular medication use, educational attainment, smoking status, alcohol consumption, and BMI, for sBP and dBP respectively. 95% confidence intervals are also shown (light blue shading).

Results from robust linear regression models investigating the relationship between the SHARE-FI continuous score and BP SampEn are reported in Table 2. For all three models, higher dBP SampEn was associated with higher SHARE-FI score (*β* = 0.38 to 0.43 (per 1 SD), *p* ≤ 0.008). All models also revealed a higher disease burden/severity (as quantified by CIRS-G score) with increased SHARE-FI score (*β* = 0.19 to 0.20, *p* ≤ 0.003). In model 3(a), being a current smoker was associated with higher SHARE-FI score; however, smoking was not significant in model 3(b). The linear relationships between the SHARE-FI score and BP SampEn are also illustrated graphically in Figure 1 using margin plots from each of the models investigated. Residuals from the models, as assessed visually by normal quantile plots and standardized normal probability plots, and numerically by the skewness-kurtosis test for normality, were normally distributed. There was no collinearity between covariates in models (2) and (3), with all VIF values ≤ 1.46.

## 4. Discussion

In previous TILDA population-based studies, we found that continuous blood pressure signal entropy could provide a clinically useful marker of the physiological dysregulations that may underlie physical frailty [15,22,23,24]. The aim of the present study was to investigate whether this measure retained its utility in a pilot clinical sample of older patients aged 50 years or over attending an outpatient falls and syncope clinic. Consistent with the previous TILDA study [15], entropy in dBP was independently associated with physical frailty in this independent clinical validation sample. However, we did not confirm the same for entropy in sBP.

Signal entropy calculated in short length BP data during rest, as used in this study, may quantify the systemic dysregulation of the cardiovascular system that underlies physical frailty. There are several potential physiological causes for this dysregulation. An increase of sympathetic activity and/or modulation directed to the heart and/or blood vessels, as previously described in abnormal ageing states [28], could produce higher levels of disorder in resting state BP and heart rate data, as detected by signal entropy. Other potential causes of increased dysregulation, and therefore potentially higher levels of entropy in cardiovascular data, include changes in arterial structure (e.g., increased stiffness, decreased compliance, and endothelial dysfunction), modified cardiac reserve, impaired baroreflex control, and increased collagen/stiffness in the left ventricle resulting in reduced diastolic filling [29,30].

The reasons as to why dBP SampEn, and not sBP SampEn, were associated with physical frailty in the present study could be both physiological and statistical. Physiologically, sBP is more related with peripheral end-organ perfusion [31], whilst dBP is more related to the perfusion of the heart muscle during its relaxation [32]. A previous study showed that both sBP and dBP pathology independently influenced the risk of adverse cardiovascular events in community-dwelling adults [33]. In our clinical sample of older FASU attendees, the prevalence of one or more cardiovascular conditions was 64%, and this may explain a higher association of dBP entropy with frailty status. The diastolic phase is also more sensitive to changes in heart rate [34] and so a measure of diastolic BP entropy may also capture an effect of heart rate entropy. On the other hand, the lack of association with sBP could be due to the lack of statistical power in this pilot sample, as previously in the much larger TILDA cohort we observed smaller effect sizes for sBP entropy versus dBP entropy, with regards associations with physical frailty [15].

Other previous studies investigating the associations between cardiovascular signal entropy and frailty status have reported contradictory results, with some reporting higher levels of entropy in cardiovascular data to be associated with worse frailty status [35,36], others conversely report lower entropy values to be associated with worse frailty status [37,38], while another study did not find any significant differences between frail, pre-frail, and non-frail groups [39]. Differences in the methodologies employed, such as frailty operationalization chosen, timeframes examined and preprocessing approaches, likely account for these discrepancies between studies, and make comparison with the present work difficult. In a 2008 study of 389 community-dwelling women, Chaves et al. reported lower approximate entropy in heart rate data to be associated with being frail by physical phenotype [37]. However, there are some important caveats to be considered when interpreting the results of that study, such as the fact that frailty status was dichotomous (frail: ’*yes*’ or ’*no*’), and that the continuous entropy measure was also dichotomized into ‘*low*’ and ‘*high*’ entropy, with ‘*low entropy*’ defined as bottom three quartiles of data. Additionally, 2 to 3 h of heart rate data was used in that study, versus the comparatively very short length (1 min) blood pressure data used in the present work, and as such these different approaches may be quantifying different physiological processes (i.e., long-term heart rate variability versus short-term impaired cardiovascular autonomic control). Using similar methodologies (i.e., operationalizing the physical frailty phenotype and calculating approximate entropy in heart rate signals), but with shorter data length (10 min) and entropy analyzed as a continuous variable, in 2014 Takahashi et al. reported higher entropy in pre-frail and frail groups, versus non-frail [36]. Another study in 2015 of 23 older women found no significant difference in SampEn between groups, despite finding significant differences between frail and non-frail/pre-frail groups (as per physical frailty definition) for more traditional variability measures (low- and high-frequency bands for the cardiac interval series) [39]. Contradictory to the results of the present work, a 2020 study by Rangasamy et al. reported lower multiscale entropy in BP data for frail versus non-frail groups [38]; however, in that study frailty was operationalized using a very different approach, compared with the present work, with the patient being categorized as frail if one or more of the following criteria was present: *age > 70 years, preoperative BMI < 18.5, hematocrit < 35%, albumin < 3.4 g/dL, or creatinine > 2.0 mg/dL*.

The approach to BP entropy calculation presented herein has been specifically designed for easy use in a clinical setting. All BP measures were non-invasive, non-ionizing, and were performed using a type of non-invasive continuous hemodynamic monitoring device that is generally available in specialized clinics. The short data length (1 min), and the fact that BP can be measured during supine rest, make the approach feasible and practical for use in a busy clinic. Entropy provides a single-value measure allowing for easy use by clinicians, which could theoretically be calculated and displayed at the bedside on the measurement device itself, though this will require further work with regards algorithm optimization and methodological developments for real-time data processing. Future work investigating how these measures vary over time would also be of interest, as perhaps BP entropy may provide an ‘early warning’ measure for monitoring transitions to more adverse frailty states, and it can be difficult for clinicians to identify with the naked eye the often slow but insidious process of frailty progression in individual patients. Additional future work will also be needed to come to a consensus as the most appropriate pre-processing and entropy calculation methodology to employ, and this would potentially allow for the establishment of age- and sex-specific normative values to use as reference guidelines in a clinical setting.

There are additional limitations which should be considered when interpreting the results of the present work. While the BP SampEn variables are objectively measured, a number of the covariates, as well as certain components of the SHARE-FI score were self-reported. Additionally, the SHARE-FI operationalization used herein does not include objective measures of mobility; however, in our same FASU context it was previously shown that SHARE-FI significantly captured gait speed in this same clinical setting, adding to its validity [25]. Furthermore, this pilot clinical validation work is of cross-sectional nature and further work will consider longitudinal patient outcomes as previously conducted in the TILDA population-based cohort [22,23,24].

## 5. Conclusions

Results from this clinical replication study demonstrated a significant and independent association between diastolic blood pressure signal entropy and physical frailty status in a pilot sample of patients aged 50 years or older attending a falls and syncope clinic. These results mirror those we previously obtained from a large population-based cohort and demonstrate the potential that a simple automated measure of blood pressure signal entropy at rest could provide a clinically useful indicator of the early physiological dysregulations that underlie physical frailty in older adults.

## Figures and Tables

**Table 1:** Characteristics of the study sample. Continuous variables are reported as the mean value, with standard deviation (SD) and ranges. Dichotomous variables are reported as count and percentage. *p*-values are given for the differences between non-frail, pre-frail, and frail groups, from Spearman’s rank correlation coefficients (continuous variables) and chi-square for trend tests (dichotomous variables).

	Full-Cohort (*n* = 100)	Non-Frail (*n* = 51)	Pre-Frail (*n* = 30)	Frail (*n* = 19)	*p*
**SHARE-FI score**	0.98 (SD: 1.58, range: [−1.80–5.47])	−0.23 (SD: 0.68, range: [−1.80–1.10])	1.53 (SD: 0.67, range: [0.36–2.79])	3.37 (SD: 0.98, range: [2.18–5.47])	**≤0.001**
**sBP SampEn**	0.20 (SD: 0.07, range: [0.04–0.35])	0.16 (SD: 0.08, range: [0.04–0.36])	0.20 (SD: 0.06, range: [0.08–0.31])	0.20 (SD: 0.08, range: [0.04–0.35])	0.730
**dBP SampEn**	0.18 (SD: 0.08, range: [0.04–0.45])	0.17 (SD: 0.07, range: [0.04–0.41])	0.17 (SD: 0.07, range: [0.06–0.41])	0.21 (SD: 0.09, range: [0.06–0.45])	**0.035**
**Age (years)**	69.9 (SD: 10.8, range: [50–93])	67.9 (SD: 10.2, range: [50–89])	73.2 (SD: 10.9, range: [50–93])	70.3 (SD: 11.4, range: [51–87])	0.164
**Sex [% (*n*)]** Female	55.0% (55)	43.1% (22)	66.7% (20)	68.4% (13)	**0.026**
**CIRS-G score**	6.95 (SD: 4.00, range: [0–20])	5.73 (SD: 3.89, range: [0–20])	7.50 (SD: 4.06, range: [0–14])	9.37 (SD: 2.93, range: [2–16])	**≤0.001**
**1 or more CV diseases ^b^ [% (*n*)]** Yes	64% [64]	60.8% [31]	73.3% [22]	57.9% [11]	0.898
**CV Medication ^a^ [% (*n*)]** Yes	49.0% (49)	43.1% (22)	63.3% (19)	42.1% (8)	0.665
**Education [% (*n*)]** Secondary/Higher	79.0% (79)	86.3% (44)	73.3% (22)	68.4% (13)	0.071
**Smoking [% (*n*)]** Current	11.0% (11)	8.9% (3)	10.0% (3)	26.3% (5)	**0.023**
**Alcohol (units per week)**	8.20 (SD: 15.6, range: [0–100])	7.35 (SD: 9.51, range: [0–37])	8.40 (SD: 17.5, range: [0–84])	10.2 (SD: 24.4, range: [0–100])	0.228
**BMI (kg m^−2^)**	26.4 (SD: 4.76, range: [17.6–39.2])	26.9 (SD: 3.84, range: [18.2–38.5])	24.6 (SD: 4.96, range: [17.6–38.2])	28.0 (SD: 5.96, range: [18.5–39.2])	0.443

^a^ Cardiovascular medications (coded using the Anatomical Therapeutic Chemical Classification (ATC)): anti-arrhythmics (ATC C01), anti-hypertensives (ATC C02), diuretics (ATC C03), vasodilators (ATC C04), beta-blocking agents (ATC C07), calcium channel blockers (ATC C08), or agents acting on renin-angiotensin system (ATC C09). ^b^ Cardiovascular diseases: hypertension, hypercholestrolaemia, ischaemic heart disease, coronary artery disease, stroke, transient ischaemic attack, atrial fibrillation or other arrythmia, heart failure, or valvular heart disease. Abbreviations: SD, standard deviation; SHARE-FI, Survey of Health, Ageing and Retirement in Europe Frailty Instrument for primary care; sBP, systolic blood pressure; dBP, diastolic blood pressure; SampEn, sample entropy; CIRS-G, Cumulative Illness Rating Scale-Geriatric; CV, cardiovascular; BMI, body mass index.

**Table 2:** Results from robust linear regression models investigating the relationship between SHAR-FI score and BP sample entropy (SampEn). Three sets of models were used: Model 1, unadjusted (bivariate); Model 2, adjusted for basic demographics and health measures (age, sex, CIRS-G score, and cardiovascular medication use); and Model 3, additionally adjusted for lifestyle socioeconomic factors (age, sex, CIRS-G score, cardiovascular medication use, educational attainment, smoking status, alcohol consumption, and BMI). All SampEn measures were standardized (z-scores) prior to analyses. Results from absolute coefficients are reported as point estimates (*β* coefficients) in appropriate units and presented with 95% CI. Statistical significance was set at *p* < 0.05.

Model	Measure	*β*	*p*	95% CI
Model 1(a)	sBP SampEn (per 1 SD)	0.05	0.734	−0.22 to 0.32
Model 1(b)	dBP SampEn (per 1 SD)	0.43	**0.004**	0.14 to 0.72
Model 2(a)	sBP SampEn (per 1 SD)	0.13	0.305	−0.12 to 0.38
	Age (per 1 year)	0.01	0.608	−0.02 to 0.04
	Sex (female)	0.19	0.472	−0.33 to 0.70
	CIRS-G score	0.20	**0.001**	0.09 to 0.32
	CV Medication ^a^ (yes)	0.03	0.930	−0.61 to 0.67
Model 2(b)	dBP SampEn (per 1 SD)	0.38	**0.008**	0.10 to 0.66
	Age (per 1 year)	0.01	0.478	−0.02 to 0.04
	Sex (female)	0.18	0.482	−0.32 to 0.68
	CIRS-G score	0.19	**0.001**	0.08 to 0.30
	CV Medication ^a^ (yes)	0.05	0.885	−0.57 to 0.66
Model 3(a)	sBP SampEn (per 1 SD)	0.12	0.365	−0.15 to 0.39
	Age (per 1 year)	0.01	0.553	−0.03 to 0.05
	Sex (female)	0.18	0.517	−0.37 to 0.73
	CIRS-G score	0.20	**0.003**	0.07 to 0.33
	CV Medication ^a^ (yes)	0.14	0.661	−0.50 to 0.78
	Education (ref Primary) Secondary/Tertiary	0.05	0.914	−0.78 to 0.87
	Smoking (ref Never/Past) Current	0.93	**0.045**	0.02 to 1.84
	Alcohol (per unit per week)	−0.01	0.238	−0.03 to 0.01
	BMI (per 1 kg m^−2^)	0.001	0.990	−0.07 to 0.08
Model 3(b)	dBP SampEn (per 1 SD)	0.39	**0.008**	0.11 to 0.67
	Age (per 1 year)	0.01	0.532	−0.02 to 0.05
	Sex (female)	0.16	0.545	−0.37 to 0.69
	CIRS-G score	0.19	**0.003**	0.07 to 0.32
	CV Medication ^a^ (yes)	0.17	0.588	−0.45 to 0.79
	Education (ref Primary) Secondary/Tertiary	0.04	0.930	−0.74 to 0.81
	Smoking (ref Never/Past) Current	0.78	0.070	−0.07 to 1.63
	Alcohol (per unit per week)	−0.01	0.154	−0.03 to 0.01
	BMI (per 1 kg m^−2^)	−0.02	0.675	−0.09 to 0.06

^a^ Cardiovascular medications (coded using the Anatomical Therapeutic Chemical Classification (ATC)): anti-arrhythmics (ATC C01), anti-hypertensives (ATC C02), diuretics (ATC C03), vasodilators (ATC C04), beta-blocking agents (ATC C07), calcium channel blockers (ATC C08), or agents acting on renin-angiotensin system (ATC C09).

## Data Availability

Not applicable.

## Appendix A

Sex-specific weightings for each of the five frailty operationalization variables are given below, along with sex-specific cut-off for ‘non-frail’, ‘pre-frail’, and ‘frail’ categorization, as derived from the SHARE study [10].

### Appendix A.1. Female

- Fatigue (*w*_fatigue_) = 0.4088
- Weight loss (*w*_weightloss_) = 0.3325
- Grip strength (*w*_gripstrength_) = −0.4910
- Weakness (*w*_weakness_) = 0.6012
- Low activity (*w*_lowactivity_) = 0.4818

### Appendix A.2. Male

- Fatigue (*w*_fatigue_) = 0.3762
- Weight loss (*w*_weightloss_) = 0.3130
- Grip strength (*w*_gripstrength_) = −0.4653
- Weakness (*w*_weakness_) = 0.6146
- Low activity (*w*_lowactivity_) = 0.4680

### Appendix A.3. Female SHARE-FI Score Cut-Offs for ‘Non-Frail’, ‘Pre-Frail’, and ‘Frail’ Categorization

- Non-frail: SHARE-FI score < 0.3151
- Pre-frail: SHARE-FI score 0.3151 to 2.1301
- Frail: SHARE-FI score > 2.1301

### Appendix A.4. Male SHARE-FI Score Cut-Offs for ‘Non-Frail’, ‘Pre-Frail’, and ‘Frail’ Categorization

- Non-frail: SHARE-FI score < 1.2119
- Pre-frail: SHARE-FI score 1.2119 to 3.0053
- Frail: SHARE-FI score > 3.0053
